# The Brief Case: Incidental finding of *Brucella abortus* bacteremia in a patient with urinary tract infection

**DOI:** 10.1128/jcm.01381-23

**Published:** 2024-04-10

**Authors:** Vincent A. Streva, Jacqueline Weinstein, Cherylann Jankowski-Romano, Nonso Osakwe, Scott Duong, Stefan Juretschko, Jamie K. Lemon

**Affiliations:** 1 Department of Pathology and Laboratory Medicine, Northwell Health, New York, New York, USA; 2 Donald and Barbara Zucker School of Medicine at Hofstra/Northwell, New York, New York, USA; 3 Department of Medicine, Northern Westchester Hospital, Northwell Health, New York, New York, USA; Pattern Bioscience, Austin, Texas, USA

**Keywords:** *Brucella*, select agent, laboratory acquired infections

## CASE

A previously healthy 17-year-old female with no prior medical history presented to the emergency department (ED) of a community hospital with 2-day history of fever, back pain, headache, and dysuria. The patient denied vomiting, diarrhea, abdominal pain, chest pain, or shortness of breath. Vital signs were unremarkable and there were no significant findings on physical exam. Blood work was unremarkable except for elevated C-reactive protein (104 mg/L; Reference Range: <4 mg/L). Urine was collected for urinalysis and showed positive urine nitrites, large leukocyte esterase, and >100 white blood cells per high power field. CT scan of the patient's abdomen and pelvis demonstrated findings consistent with pyelonephritis. Urine and blood cultures (two sets) were submitted to the laboratory. Following hospital admission and administration of IV ceftriaxone and supportive fluids, patient reported improved symptoms. After an unremarkable 1-day hospital stay, the patient was discharged on twice-daily cefdinir (300 mg PO for 9 days). Urine culture results reported shortly after discharge showed >100,000 CFU/mL of β-lactam-susceptible *Escherichia coli*.

**Fig 1:**
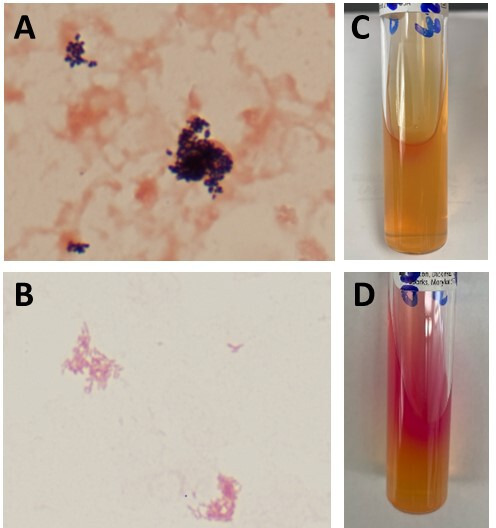
*Brucella abortus* culture findings. Gram stain results from the positive blood culture bottle initially showed retention of crystal violet stain and appeared Gram-positive (Fig. 1A). Repeat Gram staining demonstrated small pleomorphic Gram-negative rods (Fig. 1B), consistent with *Brucella* species. The isolate recovered from subculture was weakly positive for urease at 3 hours of incubation (Fig. 1C) and strongly positive after 20 hours of incubation (Fig. 1D).

Two days after discharge, one aerobic blood culture bottle flagged positive after ~64 hours of incubation. Gram stain of the positive bottle was initially reported as Gram-positive rods (Fig. 1A), which were interpreted by the provider as probable contamination. Multiplex blood culture identification PCR (BioFire Diagnostics, BioFire Diagnostics, Salt Lake City, UT) was negative. Direct MALDI-ToF analysis from the blood culture bottle yielded no definitive identification but had a low-score match (1.42) for *Brucella melitensis* using the Bruker Security-Relevant (SR) library (Bruker Daltonics, Billerica, MA). This prompted repeat Gram stain, which demonstrated small pleomorphic Gram-negative rods (Fig. 1B). The laboratory performed additional multiplex PCR testing (BioFire FilmArray BioThreat Research Use Only Panel, BioFire Defense, Salt Lake City, UT) directly from the positive blood culture bottle, which was positive for *Brucella* species. The New York State Department of Health and clinical team were notified.

The patient was instructed to return to the ED for consultation with Infectious Disease and additional laboratory testing. Upon return, the patient was well-appearing and denied any symptoms of illness. A more detailed patient history was obtained. The patient immigrated to New York from Guatemala 1 year previously. She endorsed no recent travel and no consumption of unpasteurized dairy or uncooked meat, and denied contact with animals, hunting, or visits to slaughterhouses. The patient was prescribed a 6-week course of doxycycline (100 mg, twice daily) and rifampin (600 mg, daily).

In the laboratory, the initial positive blood culture bottle grew small white colonies with a shiny surface on blood and chocolate agar plates after overnight incubation at 35°C. Colony Gram stain showed tiny Gram-negative coccobacilli consistent with those seen in the initial positive blood culture. The isolate was oxidase positive and urease positive at 3 hours (weakly) and 20 hours (more strongly) (Fig. 1C and D). Based on these findings, the laboratory was unable to rule out *Brucella* species and the isolate was forwarded to the New York State Department of Health Wadsworth Center Laboratory. Targeted PCR identified the isolate as *Brucella abortus*. A remnant serum specimen from the patient was tested for total antibodies against *Brucella abortus* using the standard tube agglutination test and was positive with a titer of 1:160, suggestive of current or recent infection. Subsequent blood cultures resulted in no growth after 5 days of incubation, and the patient was lost to follow-up after discharge.

## DISCUSSION

Brucellosis is a zoonotic bacterial infection caused by Gram-negative bacteria of select species within the *Brucella* genus that can cause life-threatening illness. Brucellosis is classically characterized by undulating fevers, sweating, and migratory arthralgia and myalgia, but often presents as non-specific, influenza-like illness, or as sub-acute or chronic illness (1–3). The three *Brucella* species most frequently associated with human infection are *B. melitensis, B. abortus*, and *B. suis (1–3*). While there are additional *Brucella* species, including the controversial recent re-classification of the *Ochrobactrum* genus into the *Brucella* genus, these species are infrequent human pathogens and typically do not cause significant human disease (1–4).

Cases of brucellosis in the United States are uncommon, with 108 reported cases in 2022 (2). However, globally, there are over 500,000 cases reported annually, predominantly in developing countries where *Brucella* is endemic (5).

Human infections are most often acquired through consumption of unpasteurized dairy products or contact with fluids or tissues of infected livestock (5). In addition to zoonotic infection, cases of brucellosis have been described in clinical laboratory workers following exposure to *Brucella* in routine microbiology culture (6). Direct person-to-person transmission is rare (7).

The gold standard laboratory diagnosis of brucellosis is made by the isolation of *Brucella* species from microbiology cultures, particularly blood or bone marrow cultures. Historic recommendations for the isolation of *Brucella* species from blood cultures included extended incubation times; however as this case illustrates, using modern blood culture instrumentation, *Brucella* isolates can be recovered within the standard 5-day incubation period. While isolation of *Brucella* species in culture is definitive, the most frequent means of diagnosis is serologic testing for antibodies against *Brucella* species. The most widely used serological assay for *Brucella* is the serum agglutination test (SAT), which detects total antibodes against brucellar smooth lipopolysaccharide and is reactive against *B. melitensis, B. abortus,* and *B. suis*. Of note, the SAT does not react with *B. canis* or the *B. abortus* RB51 vaccine strain due to their production of rough lipopolysaccharide (8). SAT titers of ≥1:160 are diagnostic for *Brucella* if accompanied by symptoms consistent with brucellosis. In patients from regions where *Brucella* species are endemic, the SAT may have reduced specificity and a higher titer cutoff (typically 1:320 or greater) is used. Enzyme-linked immunosorbent assays (ELISAs) are also available for the serological diagnosis of brucellosis; however, these assays may lack specificity when compared to SAT. Because false negative *Brucella* IgM results may occur, if ELISA testing is conducted, both IgM and IgG isotypes should be assessed (8). While serological testing for brucellosis remains diagnostically useful, providers and laboratories should be aware of potential cross-reactions with non-brucellosis-causing *Brucella* species or with other Gram-negative bacteria (8).

Given the widespread use of serological testing for *Brucella*, numerous cases of asymptomatic *Brucella* infection have been described based on serological evidence (9–11). Additionally, there is at least one documented case of an incidental brucellosis diagnosis in a patient with documented epidemiological risk factors (12).

Our case is notable for an incidental finding of *Brucella* bacteremia in a patient with no known exposures or risk factors for brucellosis, apart from emigration from an endemic area more than 12 months prior to presentation. In this case, because the positive *Brucella* culture finding was unexpected given the patient's clinical presentation and accompanying UTI, there was skepticism about the meaning of the culture result. Subsequent serologic testing was informative to convince the provider of the patient's *Brucella* infection. Scenarios, where patients do not present with classic signs and symptoms of *Brucella* infection, may result in increased risk to microbiology laboratory staff, who often rely on provider suspicion of brucellosis to implement enhanced laboratory precautions to prevent potential exposures.

Fortunately, in this case, the prolonged blood culture incubation time to positivity (>48 hours) prompted the microbiology technologist working up the culture to flag the culture for enhanced precautionary handling, including sealing all subculture plates with tape, alerting other laboratory staff about the culture, and flagging additional pending microbiology cultures from this patient. These actions meant the positive bottle and all subculture plates were safely worked up for identification exclusively in a biological safety cabinet. Additionally, inactivation of the sample via formic acid/acetonitrile extraction during preparation for MALDI-ToF analysis was performed within a biological safety cabinet prior to identification. As a result, there were no laboratory exposures from this culture. In instances where laboratory staff are exposed to *Brucella* species growing in culture, recommendations are for: (1) symptom watch, including fever monitoring for 24 weeks post-exposure; (2) baseline serological testing for *Brucella* antibodies, followed by serial serologic monitoring for a period of 24 weeks; and (3) post-exposure prophylaxis including doxycycline (100 mg twice daily) and rifampin (600 mg once daily) for 21 days as first-line treatment (13).

This case provides learning points that may be helpful to microbiology laboratories to identify cases of unexpected brucellosis. First, *Brucella* species are often slow-growing, as was the case for this isolate from the initial blood culture. Microbiology laboratory staff should be cautious of any growth of cultures after more than 48 hours of incubation. Second, *Brucella* species may retain crystal violet stain (14), so Gram stain of primary specimens may appear Gram-positive or Gram-variable rather than canonically Gram-negative, as was observed in this case. Finally, recent advances in technology including research use only multiplex PCR panels and MALDI-ToF databases may aid in the recognition of *Brucella* species prior to the availability of a culture isolate for utilization in the sentinel laboratory rule-out-or-refer algorithm. Access to these research use only products is helpful because *Brucella* species are not included in the FDA-cleared MALDI-ToF libraries used in many laboratories and may be misidentified by these databases as other organisms, including *Ochrobactrum* species. Specialty databases such as the Bruker RUO and SR libraries should be made available and routinely utilized in the laboratory to improve identification of *Brucella* species. Of note, access to these resources requires engagement with *in vitro* diagnostic manufacturers and may require additional approvals prior to installation in the laboratory. Additionally, implementation of these libraries for routine clinical use would require validation as a laboratory developed test (LDT), and therefore, laboratories must have access to sufficient numbers of uncommon bacterial isolates included in these databases in order to satisfy regulatory requirements for validation of LDTs. In our laboratory, the Bruker SR database and BioFire BioThreat panel are used exclusively in conjunction with the APHL/LRN/ASM rule-out or refer algorithm and results are provided to the LRN reference laboratory but are not reported clinically. Because the clinical presentation of brucellosis is often non-specific and may have a long incubation period, it can be challenging for physicians to recognize brucellosis and include it in their differential diagnosis. In these cases, laboratory tools such as those discussed above can be particularly helpful when the laboratory has not been notified of the potential threat of isolation of *Brucella* species.

## SELF-ASSESSMENT QUESTIONS

1. Which of the following *Brucella* species does not typically cause human brucellosis?
  - *Brucella abortus*
  - *Brucella canis*
  - *Brucella melitensis*
  - *Brucella suis*
2. Which of the following laboratory findings is inconsistent with *Brucella* spp.?
  - Oxidase positive
  - Pleomorphic Gram-negative coccobacilli
  - Urease negative
  - Growth on blood and chocolate agar media but demonstrate no growth or poor growth on MacConkey agar media
3. Following a laboratory exposure to *Brucella* in culture, which of the following should not be among the recommended actions taken by laboratories?
  - Evaluate baseline serology and subsequent serial serological testing in exposed employees for *Brucella* antibodies for a period of 24 weeks
  - Offer post-exposure prophylaxis using doxycycline and rifampin for a period of 21 days
  - Initiate regular symptom monitoring, including self-fever checks, for a period of 24 weeks
  - Perform regular blood cultures on exposed individuals for a period of 24 weeks

## ANSWERS TO SELF-ASSESSMENT QUESTIONS

1. Which of the following *Brucella* species does not typically cause human brucellosis?
  - *Brucella abortus*
  - *Brucella canis*
  - *Brucella melitensis*
  - *Brucella suis*

Answer: b – *Brucella canis*. There are three *Brucella* species that cause human brucellosis: *B. melitensis, B. abortus,* and *B. suis. Brucella canis* may cause mild symptomatic illness in people and is commonly identified in people who have close contact with dogs. The recently reclassified *Ochrobactrum* genus are rare human pathogens and do not cause brucellosis.

- Which of the following laboratory findings is inconsistent with *Brucella* spp.?
  - Oxidase positive
  - Pleomorphic Gram-negative coccobacilli
  - Urease negative
  - Growth on blood and chocolate agar media but demonstrate no growth or poor growth on MacConkey agar media

Answer: c. Members of the *Brucella* genus are nearly universally rapidly urease positive, with the exception of *B. ovis*, which is urease negative but is not known to cause human illness. Both oxidase and urease testing are part of the APHL/LRN/ASM rule-out algorithm for *Brucella*. Notably, though they are Gram-negative coccobacilli, *Brucella* spp. may retain crystal violet stain and can appear as Gram-variable or Gram-positive. *Brucella* species grow slowly on blood and chocolate agars and either do not grow or grow poorly on MacConkey agar.

- Following a laboratory exposure to *Brucella* in culture, which of the following should not be among the recommended actions taken by laboratories?
  - Evaluate baseline serology and subsequent serial serological testing in exposed employees for *Brucella* antibodies for a period of 24 weeks
  - Offer post-exposure prophylaxis using doxycycline and rifampin for a period of 21 days
  - Initiate regular symptom monitoring, including self-fever checks, for a period of 24 weeks
  - Perform regular blood cultures on exposed individuals for a period of 24 weeks

Answer: d. Regular blood culture monitoring of exposed individuals is not recommended. For high-risk exposures, Centers for Disease Control and Prevention recommendations include regular symptom check including fever monitoring, serological monitoring for *Brucella* antibodies at baseline and up to 24 weeks, and post-exposure prophylaxis with a regimen of doxycycline and rifampin for a period of 24 weeks. For patients with contraindications to first-line therapy, trimethoprim-sulfamethoxazole in addition to another appropriate antimicrobial should be considered; treatment with a single antibiotic (monotherapy) is not recommended.

**TAKE-HOME POINTS**

• Brucellosis is a zoonotic bacterial infection caused by three species of *Brucella* bacteria (*B. melitensis*, *B. abortus*, and *B. suis*).

• Brucellosis typically presents as undulating fevers, sweating, and migratory arthralgia and myalgia, but often appears as a non-specific, influenza-like illness, or as sub-acute chronic illness.

• Diagnosis of brucellosis is not only made by recovery of *Brucella* species bacteria from cultures (most often blood cultures) but also can be diagnosed through serologic testing.

• Treatment of brucellosis includes a 6-week course of doxycycline and rifampin.

• Laboratories should be aware that they cannot rely on physician suspicion of brucellosis and should implement safety measures in the laboratory for the handing of organisms with growth characteristics consistent with *Brucella* species.
